# Combined Effects of Chronic Kidney Disease and Nonalcoholic Fatty Liver Disease on the Risk of Cardiovascular Disease in Patients with Diabetes

**DOI:** 10.3390/biomedicines10061245

**Published:** 2022-05-26

**Authors:** Goh-Eun Chung, Kyungdo Han, Kyu-Na Lee, Eun-Ju Cho, Jung-Ho Bae, Sun-Young Yang, Su-Jong Yu, Seung-Ho Choi, Jeong-Yoon Yim, Nam-Ju Heo

**Affiliations:** 1 Department of Internal Medicine, Healthcare Research Institute, Gangnam Healthcare Center, Seoul National University Hospital, Seoul 06236, Korea; gohwom@snu.ac.kr (G.-E.C.); bjh@snuh.org (J.-H.B.); syyang@snuh.org (S.-Y.Y.); cshmd@snuh.org (S.-H.C.); yjy@snuh.org (J.-Y.Y.); 2 Department of Statistics and Actuarial Science, The Soongsil University, Seoul 06591, Korea; hkd917@naver.com (K.H.); rbskg8274@naver.com (K.-N.L.); 3 Department of Internal Medicine, Seoul National University Hospital, Seoul 03080, Korea; creatioex@gmail.com (E.-J.C.); sujongyu@gmail.com (S.-J.Y.)

**Keywords:** chronic kidney disease, fatty liver, myocardial infarction, stroke, type 2 diabetes

## Abstract

Background: We investigated the combined effect of chronic kidney disease (CKD) and nonalcoholic fatty liver disease (NAFLD) on the risk of cardiovascular disease (CVD) in patients with type 2 diabetes. Methods: Data were obtained from the Korean National Health Insurance Service. Patients with diabetes who participated in health screenings from 2009 to 2011 were included. The fatty liver index (FLI) was used as a surrogate marker for NAFLD. Results: During a mean follow-up of 6.9 years, 40,863 incidents of myocardial infarction (MI), 58,427 strokes, and 116,977 deaths were reported in 1,607,232 patients with type 2 diabetes. After adjusting for conventional risk factors, patients with CKD and NAFLD showed the highest risk of MI and stroke (hazard ratio (HR) = 1.49; 95% confidence interval (CI): 1.42–1.57 and stroke, HR = 1.48; 95% CI: 1.41–1.54, respectively) compared with those without either CKD or NAFLD. Both overall and cardiovascular mortality were highest in the CKD/NAFLD group compared with other groups (HR = 2.00; 95% CI: 1.94–2.06, and HR = 2.20; 95% CI: 2.07–2.35, respectively). Advanced liver fibrosis was significantly associated with an increased risk of CVD in patients with NAFLD. Proteinuria was significantly associated with incidence of CVD events in patients with CKD. Conclusions: The combination of CKD and NAFLD was associated with an increased risk of CVD and mortality in patients with type 2 diabetes. Close monitoring and appropriate management of CKD and NAFLD may be warranted to prevent CVD in these patients.

## 1. Introduction

Nonalcoholic fatty liver disease (NAFLD) is the most common cause of chronic liver disease worldwide; it has a prevalence of up to 20–30% globally [1,2]. Type 2 diabetes mellitus (T2DM) is a well-established risk factor for NAFLD, in addition to obesity, dyslipidemia, and metabolic syndrome. The prevalence of NAFLD in patients with T2DM is estimated at 56–60%, more than two-fold higher than in the general population [3,4]. In patients with T2DM, NAFLD is associated with an increased incidence of cardiovascular disease (CVD) and overall mortality [4,5].

Chronic kidney disease (CKD) is a global public health problem that affects 10–15% of the world’s population, with diabetes becoming the leading cause of CKD. Approximately 40% of patients with diabetes also have CKD [6], which is associated with a high mortality rate [7,8,9]. In patients with T2DM, CKD increases the overall risk of CVD, which is a leading cause of morbidity and mortality in these patients [10].

Previous studies have suggested an association between NAFLD and CKD [11,12], and both diseases are associated with a high risk of CVD and mortality [13,14,15]. Thus, a possible synergistic increase in cardiovascular risk as a result of the combination of NAFLD and CKD is suggested. However, no studies have examined the combined effects of NAFLD and CKD on CVD risk in patients with T2DM. Assessing the combined risk of NAFLD and CKD for CVD risk may be helpful in establishing a treatment strategy for patients with T2DM. Therefore, we aimed to investigate the combined effects of NAFLD and CKD on CVD risk, including increases in myocardial infarction (MI), stroke, and death in patients with T2DM using nationally representative data from the Korean population.

## 2. Research Design and Method

### 2.1. Data Source

The Korean National Health Insurance Services (NHIS) database includes data on outpatient visits, inpatient visits, procedures, and prescription medications based on their claims data. The Korean NHIS is a mandatory social insurance that covers the entire Koreans except for the lowest income Medicaid beneficiaries (approximately 3% of the population). Claims are accompanied by diagnostic data collected from International Statistical Classification of Diseases-Tenth Revision (ICD-10) codes and information on procedures, prescription drugs, patient demographics, hospital information, direct medical costs of both inpatient and outpatient care, and dental services [16]. NHIS provides biennial health screenings to subjects who are aged 40 and above or employees. This regular health checkup includes anthropometric measurements, laboratory examinations, and self-administered health questionnaires on lifestyle behaviors (smoking, alcohol consumption, physical activity, etc.). Questionnaires were given to participants at all screening visits before the examination and providers were regularly checked for their quality for reimbursement [17].

### 2.2. Ethics Statement

This study was approved by the Institutional Review Board of Seoul National University Hospital (IRB No. E-2110-106-1264) and Soongsil University (IRB No. SSU-202003-HR-201-01). Anonymized information was used for analyses; therefore, informed consent was not required. The database is open to all researchers whose study protocols are approved by the official review committee.

### 2.3. Study Population

Patients with T2DM were defined as having either at least one claim per year for a prescription for an antidiabetic medication under ICD-10 codes E11–14 from the insurance claims data or a fasting plasma glucose level greater than or equal to 126 mg/dL during the health examination without a prescription for insulin and/or at least one oral hypoglycemic agent (OHA) [18]. OHAs included metformin, sulfonylurea, meglitinide, thiazolidinedione, a dipeptidyl peptidase-4 inhibitor, and α-glucosidase inhibitor [19].

Among 2,745,638 patients with T2DM (age ≥ 20 years) who participated in health screenings from 2009 to 2012, patients who met the following criteria were excluded from the study: heavy alcohol consumption (≥30 g for male, and ≥20 g for female of alcohol/day, n = 283,167), previous diagnosis of liver cirrhosis (K74) or hepatitis (B15–B19) (n = 427,338), history of MI (I21, I22, n = 81,167) or stroke (I63, I64) before the index year (n = 197,940), or missing information (n = 99,101). We ascertained outcome events after a lag of 1 year, and those with outcome events within 1 year were excluded (n = 49,693). Finally, a total of 1,607,232 subjects with T2DM were included in the study.

### 2.4. Measurements

Standardized self-reported questionnaires were used to collect data at the time of enrollment as described previously [20,21]. Smoking status was classified as: never-smokers, ex-smokers, and current smokers. Participants reported alcohol consumption frequency (as 0, 1–2, 3–4, and 5–7 times per week) and the amount of alcohol consumption as the number of glasses consumed per occasion [22]. Participants were then categorized according to their daily average amount of alcohol consumption: none and mild-to-moderate (<30 g alcohol/day for males and <20 g alcohol/day for females). Regular physical activity was defined as engaging in physical activity of a moderate to vigorous intensity for three or more days per week. Household income was dichotomized at the lowest 20%.

During the national health screening, height, weight, and waist circumference (WC) were evaluated by healthcare professionals. Body mass index (BMI) was calculated by weight/height squared (kg/m^2^). Systolic and diastolic blood pressure was measured in a seated position after at least 5 min of rest. Participants were considered as having hypertension if either systolic (≥140 mm Hg) or diastolic (≥90 mm Hg) blood pressure was increased; and/or prescription for an anti-hypertensive agent linked to the hypertension ICD-10 diagnosis codes (I10–I13 or I15). Dyslipidemia was defined as a total cholesterol level greater than or equal to 240 mg/dL and/or prescription for a lipid-lowering agent that associated with an ICD-10 claim code (E78) [23].

Blood specimens were collected after overnight fasting. Serum fasting glucose, triglycerides, total cholesterol, high-density lipoprotein (HDL) cholesterol, creatinine, and liver function tests including aspartate transaminase (AST), alanine transaminase (ALT), and gamma-glutamyl transferase (GGT) levels were measured. CKD was defined as an estimated glomerular filtration rate (eGFR) less than 60 mL/min/1.73 m^2^ calculated using the Modification of Diet in Renal Disease (MDRD) equation and the Chronic Kidney Disease Epidemiology Collaboration (CKD-EPI) equation [24]. The participants were tested for urine protein using the dipstick method. Proteinuria status was defined as negative, trace, or from 1+ to 4+ [25].

### 2.5. Surrogate Measurement of NAFLD and Probability of Advanced Fibrosis

Although ultrasonography is the gold standard screening technique for NAFLD in clinical practice [26], it is not employed in the NHIS mass screening program. Instead, biochemical results were used to calculate the fatty liver index (FLI) score according to the following formula [27]:

FLI = e^0.953 × ln triglycerides + 0.139 × BMI + 0.718 × ln GGT + 0.053 × WC − 15.745^/(1 + e^0.953 × ln triglycerides + 0.139^
^× BMI + 0.718 × ln GGT + 0.053 × WC − 15.745^) × 100

The FLI score ranges from 0 to 100, with less than 30 representing a low risk of a fatty liver and greater than or equal to 60 representing a high risk of a fatty liver [28]. An FLI value greater than or equal to 60 was previously reported as a surrogate marker for fatty liver and was found to accurately diagnose steatosis [27] and correlate with insulin resistance [29]. The FLI score was previously validated to detect ultrasound-diagnoses of fatty liver with the area under the receiver operating characteristics curves ranging from 0.79–0.87 in Korean populations [30,31]. Thus, we defined the NAFLD and control groups as an FLI value greater than or equal to 60 and less than 60, respectively. In addition, we used the AST/ALT ratio (AAR) as a surrogate value to define low (<0.8), middle (0.8–1.4), and high (≥1.4) probabilities of advanced fibrosis in patients with NAFLD using cut-offs of 0.8 and 1.4 [32,33].

### 2.6. Outcome Variable

The primary study endpoints were cardiovascular events including newly diagnosed MI, stroke, or overall and cardiovascular death. MI was defined as a record of ICD-10 codes I21 or I22 during hospitalization or such codes recorded at least two times previously. Stroke was defined as a record of ICD-10 codes I63 or I64 during hospitalization with claims for brain computerized tomography or magnetic resonance imaging [34].

Information on mortality and cause of death was available for all subjects in the cohort; the latter was classified according to the Korean Standard Classification of Diseases and Causes of Death (http://kssc.kostat.go.kr/ksscNew_web/ekssc/main/main.do#, accessed on 1 August 2021) based on the ICD-10, as provided by the Korean National Statistical Office. Cardiovascular death was based on the record of ICD-10 codes (I00–I99). The study population was followed from baseline to the date of the study outcomes, or until 31 December 2018, whichever occurred first.

### 2.7. Statistical Analysis

Continuous variables were expressed as the means ± standard deviations or median (interquartile range) if the distribution was skewed. Categorical variables were expressed as numbers (percentage). Comparisons of baseline characteristics were made using the independent Student’s *t* test and analysis of variance for continuous variables and the chi-square test for categorical variables.

We divided participants into four groups according to the presence of CKD or NAFLD: no CKD/no NAFLD, no CKD/NAFLD, CKD/no NAFLD, and CKD/NAFLD, and performed inverse probability of treatment weighting (IPTW) using propensity scores derived from the baseline covariates including age, sex, alcohol consumption, and smoking status to balance the weights across the four groups. Maximum absolute standardized difference < 0.1 (10%) was evaluated as a negligible difference in the baseline covariates between the study groups [35,36]. After IPTW, the risks of cardiovascular events were computed by weighted Cox proportional hazards models with IPTW.

Model 1 was adjusted for age and sex. Model 2 was adjusted as in Model 1 and additionally for lifestyle factors such as smoking status, alcohol consumption, physical activity, hypertension, dyslipidemia, income, T2DM duration, insulin use, and OHA use. Model 3 was adjusted as in Model 2 and additionally for BMI. Model 4 was adjusted as in Model 3 and additionally for statin use. Stratified analyses were performed according to age, sex, hypertension, alcohol consumption, insulin use, and T2DM duration. To test for potential effect modifications, stratified analyses were performed according to risk factor subgroups and forest plots were constructed. A *p* value less than 0.05 was considered to indicate statistical significance. The statistical analyses were performed using SAS, version 9.4 (SAS Institute Inc., Cary, NC, USA).

## 3. Results

### 3.1. Characteristics of Study Population

The baseline characteristics of each group are shown in Table 1. Compared with the no CKD/no NAFLD group, people in the CKD/no NAFLD group were older and more likely to be female (all *p* < 0.001). Additionally, subjects in the CKD/no NAFLD, no CKD/NAFLD, and CKD/NAFLD groups were more likely to have hypertension and dyslipidemia than those in the no NAFLD/no CKD group (*p* < 0.001). People in the CKD/no NAFLD and CKD/NAFLD groups were more likely to have had T2DM for a longer duration and more frequently used insulin, an oral glucose agent, or statin than those in the no NAFLD/no CKD group (*p* < 0.001). Most anthropometric and laboratory variables (including BMI, systolic/diastolic blood pressure, fasting glucose level, and HDL cholesterol level) were less metabolically favorable in the no CKD/NAFLD, CKD/no NAFLD, and NAFLD/CKD groups than in the no CKD/no NAFLD group (all *p* < 0.001). People in the CKD/NAFLD showed higher prevalence of heavy proteinuria (3 or 4+) compared to other groups (*p* < 0.001).

### 3.2. Risk of CVD According to NAFLD and CKD

**Figure 1:**
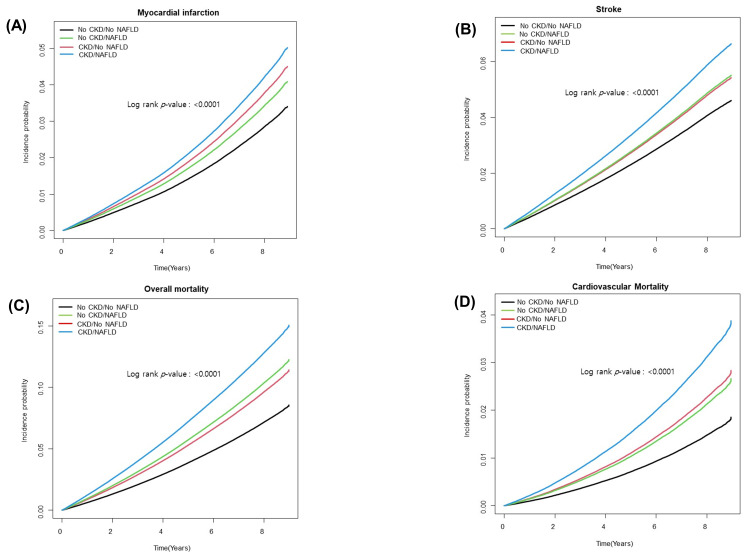
Kaplan–Meier curves showing cumulative event-free survival in patients classified into four groups based on the presence or absence of CKD and NAFLD. (**A**) Myocardial infarction, (**B**) stroke, (**C**) overall mortality, and (**D**) cardiovascular mortality. Cox proportional hazards model were used and adjusted for age, sex, income, alcohol consumption, smoking, regular exercise, income, diabetes mellitus, hypertension, dyslipidemia, DM duration, insulin use, and OHA use. Abbreviations: CI, confidence interval; CKD, chronic kidney disease; HR, hazard ratio; NAFLD, nonalcoholic fatty liver disease.

During a mean follow-up of 6.9 years, 40,863 incident cases of MI, 58,427 cases of stroke, and 116,977 deaths developed among 1,607,232 patients with T2DM. Table 2 summarizes the association of NAFLD and CKD with incidence of CVD events in patients with T2DM. In Model 1, those in the CKD/NAFLD group had the highest risk of incident MI and stroke compared to the no CKD/no NAFLD group [MI, hazard ratio (HR) = 1.56; 95% confidence interval (CI): 1.49–1.64, and stroke, HR = 1.51; 95% CI: 1.45–1.57, respectively]. In Models 2 and 3, the risk of MI and stroke were still the highest in the CKD/NAFLD group compared to the no CKD/no NAFLD group (MI, HR = 1.49; 95% CI: 1.42–1.57, and stroke, HR = 1.48; 95% CI: 1.41–1.54, respectively), which were higher than the risk in either the no CKD/NAFLD group (HR = 1.21 and HR = 1.21) or the CKD/no NAFLD group (HR = 1.33 and HR = 1.19). Overall and cardiovascular mortality were highest in the CKD/NAFLD group (HR = 2.00; 95% CI: 1.94–2.06, and HR = 2.20; 95% CI: 2.07–2.35, respectively) compared with the no CKD/NAFLD group (HR = 1.54 and HR = 1.47, respectively) and the CKD/no NAFLD group (HR = 1.41 and HR = 1.57, respectively) in Model 3. After IPTW, the highest increased risk of all cardiovascular events in the CKD/NAFLD group still remained (Table 2). When we further adjusted for statin use in Model 4, similar results were observed (Supplementary Table S1). Kaplan–Meier survival curves demonstrated that patients with both CKD and NAFLD were at a significantly higher risk for the development of CVD events (Figure 1). When applying the CKD-EPI equation in the CKD definition, the results were similar to those when the MDRD equation was applied. (Supplementary Table S2).

### 3.3. Stratified Analyses

**Figure 2:**
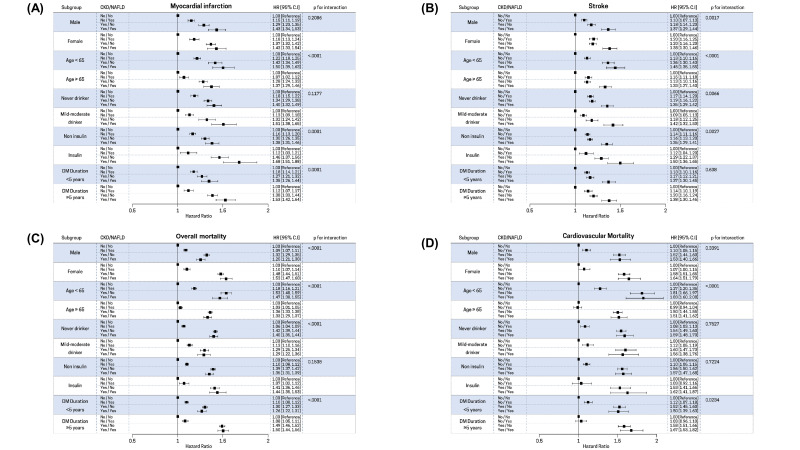
Forest plot showing the HRs of the association between CKD/NAFLD and (**A**) incidence of myocardial infarction, (**B**) stroke, (**C**) overall mortality, and (**D**) cardiovascular mortality in different subgroups: male vs. female; age < 65 vs. ≥65 years; never drinker vs. mild-moderate drinker; no-insulin vs. insulin use and DM duration < 5 years vs. DM duration ≥ 5 years. Adjusted for age, sex, income, alcohol consumption, smoking, regular exercise, income, diabetes mellitus, hypertension, dyslipidemia, DM duration, insulin use, and OHA use. HRs are represented by black squares; 95% CIs are denoted by horizontal whiskers. Abbreviations: CI, confidence interval; HR, hazard ratio; CKD chronic kidney disease; DM, diabetes; NAFLD, nonalcoholic fatty liver disease.

We performed a subgroup analysis stratified by sex (male vs. female), age (<65 years vs. ≥ 65 years), alcohol intake (none vs. mild-to-moderate drinker), insulin use (no vs. yes), and T2DM duration (<5 years vs. ≥5 years). Stratified analyses also showed generally similar associations between the presence of NAFLD/CKD and CVD events (Figure 2). The increased risk of MI in the CKD/NAFLD group was more prominent in younger subjects, insulin users, and those with a T2DM duration of at least 5 years. The risk of stroke associated with NAFLD and CKD was more prominent in females, younger subjects, alcohol drinkers, and insulin users. In addition, the association between NAFLD/CKD and overall mortality risk was more prominent in females, younger subjects, never drinkers, and those with a T2DM duration of at least 5 years. The association between NAFLD/CKD and cardiovascular mortality was more prominent in younger subjects and those with a T2DM duration of at least 5 years (Figure 2).

### 3.4. Subgroup Analysis in Subjects with NAFLD

We further explored the association between the probability of advanced fibrosis and CVD events in NAFLD patients with T2DM. Multivariate-adjusted models showed that those with a high AAR (≥1.4) were 13% (HR = 1.13; 95% CI: 1.04–1.23) and 29% (HR = 1.29; 95% CI: 1.20–1.37) more likely to experience MI and stroke in the absence of CKD. In patients with CKD, the risk of MI and stroke increased significantly by 38% (HR = 1.38; 95% CI, 1.21–1.58) and 50% (HR = 1.50; 95% CI: 1.36–1.67) in individuals with a high AAR (≥1.4) compared with those with a low AAR (<0.8) after adjusting for confounders (Table 3).

Overall and cardiovascular mortality significantly increased in those with high AAR (≥1.4) in both non-CKD and CKD groups compared to those with low AAR (<0.8). (without CKD, HR = 2.16; 95% CI: 2.06–2.25 and HR = 1.93; 95% CI: 1.73–2.15, and with CKD, HR = 2.36; 95% CI: 2.20–2.52 and HR = 2.57; 95% CI: 2.21–2.98, respectively, Table 3).

### 3.5. Subgroup Analysis in Subjects with CKD

Next, we investigated the association between the degree of proteinuria and CVD events in T2DM patients with CKD. Multivariate-adjusted models showed that those with heavy proteinuria (3 or 4+) were two-fold more likely to experience MI and stroke in the absence of NAFLD (MI, HR = 2.34; 95% CI: 2.06–2.65, and stroke, HR = 2.33; 95% CI: 2.09–2.59). In patients with NAFLD and heavy proteinuria, the risk of MI and stroke increased almost three-fold (MI, HR = 2.91; 95% CI, 2.38–3.55, and stroke, HR = 2.74; 95% CI: 2.30–3.25) compared to individuals without proteinuria after adjusting for confounders. Overall and cardiovascular mortality significantly increased in those with heavy (3 or 4+) proteinuria compared with those without proteinuria in patients with or without NAFLD (with NAFLD and heavy proteinuria, HR = 3.53; 95% CI: 3.14–3.98, with NAFLD and no proteinuria, HR=3.77; 95% CI: 2.94–4.83, without NAFLD and heavy proteinuria, HR = 2.59; 95% CI: 2.42–2.76, and without NAFLD and no proteinuria, HR = 2.81; 95% CI: 2.44–3.25). Similar trends were observed when applying IPTW (Table 4).

## 4. Discussion

In this nationwide, population-based cohort study, we found that the risk for CVD, including MI, stroke, and cardiovascular mortality, increased significantly in the CKD/NAFLD group compared to the no CKD/no NAFLD group among patients with T2DM, independent of conventional cardiovascular risk factors. The risk of CVD events was higher in patients with both CKD and NAFLD than in patients with either CKD or NAFLD alone. Advanced liver fibrosis was significantly associated with incidence of CVD events in patients with NAFLD, and the risk of CVD was higher in patients with CKD than without CKD. Furthermore, proteinuria significantly increased the risk of CVD in patients with CKD, and the risk of CVD was higher in patients with NAFLD than without NAFLD. To the best of our knowledge, this study is the first prospective, large-scale investigation reporting the combination effects of CKD and NAFLD on the risk of CVD and mortality among individuals with diabetes.

The effects of NAFLD or CKD on CVD risk in patients with T2DM have been previously shown. Patients with T2DM and NAFLD have a higher risk of CVD compared to patients with T2DM alone, suggesting a potential synergistic increase in CVD risk in patients affected by both conditions [37,38]. Consistent with previous results, the risk of CVD events, including MI, stroke, and cardiovascular mortality, increased significantly in patients with T2DM and NAFLD compared to those with T2DM without NAFLD in our study. The potential mechanism linking NAFLD and CVD in patients with T2DM includes pro-atherogenic alterations in lipid profiles, increased levels of thrombotic factors, low-grade inflammation, insulin resistance, and microbiome alterations [39].

Patients with diabetes have a higher risk of developing CKD, which has been reported to be 3.34 (95% CI: 2.27–4.93) in women and 2.84 (95% CI: 1.73–4.68) in men [40]. In addition, the presence of both T2DM and CKD causes a higher risk of cardiovascular morbidity [41] and all-cause and cardiovascular mortality [42]. Previous studies have investigated the relationship between CKD and NAFLD; NAFLD could be a driving factor for the development and progression of CKD, with underlying mechanisms including T2DM, abnormal metabolism of lipoproteins, dysbiosis of intestinal microbiota, and platelet activation [11]. A Mendelian randomization study recently demonstrated that a genetic predisposition for NAFLD was significantly associated with a reduced eGFR after adjusting for metabolic disorders, suggesting a causal reduction in kidney function by NAFLD [43].

The mechanism for the adverse metabolic effects of NAFLD in patients with T2DM may be explained by defective lipid metabolism accompanied by triglyceride deposition in the liver as a result of insulin resistance [44]. NAFLD can exacerbate insulin resistance, leading to the release of multiple mediators such as proinflammatory, pro-oxidant, and profibrogenic factors that are essential in the pathogenesis of both CKD and CVD. In addition, kidney disease may contribute directly to the increased risk of mortality by promoting cardiovascular risk factors such as hypertension, insulin resistance, oxidative stress, endothelial cell dysfunction, and inflammation [45,46].

Although we could not exactly evaluate the severity of diabetes in the study participants, we performed a subgroup analysis stratified by T2DM duration or insulin use. The risk of CVD events was higher in patients using insulin or with a long T2DM duration. CVD risk may be related to increased insulin resistance in patients with NAFLD and a long T2DM duration or use of insulin, although we could not evaluate the insulin levels.

Given the increasing prevalence of NAFLD, especially in patients with T2DM, it is essential to identify high-risk groups with an advanced form of the disease. Advanced liver fibrosis in NAFLD is associated poor clinical outcomes such as renal and cardiovascular complications in patients with T2DM [32,47]. Consistent with results from previous studies, advanced hepatic fibrosis was associated with a higher risk of all CVD events including MI, stroke, and overall and cardiovascular mortality, and the risk of CVD was higher in patients with CKD than without CKD, confirming the additive effects of hepatic fibrosis and CKD in patients with NAFLD.

In patients with T2DM, proteinuria is the earliest marker of diabetic nephropathy and cardiovascular risk [48,49]. We reported a graded increase in cardiovascular risk and mortality with increasing proteinuria in subjects with CKD. The risk of CVD associated with proteinuria was even higher in subjects with NAFLD than without NAFLD. This study is the first investigation to report the combined effects of proteinuria and NAFLD on the risk of CVD in individuals with diabetes. Therefore, assessing proteinuria using a simple urine dipstick test could provide a useful adjunct to assess risk for CVD in subjects with T2DM, CKD, and NAFLD.

## 5. Strengths and Limitations

This study is the first large-scale investigation to determine the combined effects of eGFR-based CKD and NAFLD on the risk of CVD and mortality in individuals with diabetes. In addition, we investigated the combined effects of hepatic fibrosis and CKD, and of proteinuria and NAFLD, in subgroup analyses. However, there are several limitations to this study. First, as with all epidemiological studies, it is difficult to ascertain causality. Second, although we used the Korean NHIS database, which included the entire Korean population and those who received a national health examination (66.0% in 2009) [50], there may be a selection bias in that the analysis was performed in individuals who actually underwent the national health examination. Third, although the FLI was used to screen for the presence of fatty liver among the general population, it cannot accurately quantify hepatic steatosis and differentiate simple steatosis from steatohepatitis [29]. Moreover, the biomarkers for hepatic steatosis are not as accurate in patients with diabetes as in the general population since biomarkers are not developed for the population with diabetes and some of those biomarkers rely on diabetic status and impaired blood glucose which could over- or underestimate the prevalence of NAFLD [51]. In addition, AAR, which was used as a surrogate marker of advanced fibrosis in this study, is less specific than the NAFLD fibrosis score or FIB-4. However, we could not obtain the data required for the formula in this cohort. Fourth, a urine dipstick test is less precise than a quantitative measurement of proteinuria. However, this method is preferred as an initial screening tool for the evaluation of proteinuria because of its low cost, simplicity, and ability to provide rapid point-of-care information [52]. Finally, the evaluation of kidney function and proteinuria status at a single point in time, lack of follow-up regarding a new diagnosis of T2DM or change in kidney function, and potential misclassification of the cause of death based on ICD codes could be limitations of this study.

## 6. Conclusions

The findings of this large, population-based cohort study suggest that those with both CKD and NAFLD have a significantly increased risk of CVD than those with either one in patients with T2DM. The presence of both advanced liver fibrosis and CKD additively increased the risk of CVD in patients with NAFLD. Further, proteinuria and NAFLD increased the risk of CVD additively in patients with CKD. Close monitoring and appropriate management of CKD and NAFLD may be warranted to prevent CVD in patients with T2DM.

## Figures and Tables

**Table 1:** Baseline characteristics of the study population.

	No CKD /No NAFLD	No CKD/NAFLD	CKD/No NAFLD	CKD/NAFLD	*p*-Value
Number of subjects	1,065,190	379,638	124,507	37,879	
Age, years	56.5 ± 12.1	51.9 ± 11.5	65.3 ± 11.2	62.0 ± 11.5	<0.0001
Male (%)	567,790 (53.3)	283,075 (75.6)	49,682 (39.9)	21,439 (56.6)	<0.0001
Smoking status					<0.0001
Never-smoker (%)	657,597 (61.7)	164,527 (43.3)	90,663 (72.8)	22,810 (60.2)	
Ex-smoker (%)	170,467 (16.0)	78,392 (20.7)	18,091 (14.5)	7351 (19.4)	
Current smoker (%)	237,126 (22.3)	136,719 (36.0)	15,753 (12.7)	7736 (20.4)	
Alcohol consumption					<0.0001
None (%)	680,576 (63.9)	168,735 (44.5)	97,478 (78.3)	24,336 (64.2)	
Mild-to-moderate (%)	384,614 (36.1)	210,903 (55.6)	27,032 (21.7)	13,561 (35.8)	
Regular physical activity (%)	229,673 (21.6)	66,218 (17.4)	25,657 (20.6)	6911 (18.2)	<0.0001
BMI, kg/m^2^	23.9 ± 2.7	28.2 ± 3.4	24.1 ± 2.7	28.4 ± 3.5	<0.0001
Waist circumference (cm)	82.0 ± 7.2	93.0 ± 8.6	83.0 ± 7.3	94.2 ± 8.3	<0.0001
SBP (mmHg)	127.1 ± 15.5	132.1 ± 15.4	129.8 ± 16.6	132.9 ± 16.4	<0.0001
DBP (mmHg)	77.9 ± 9.9	82.3 ± 10.3	77.6 ± 10.3	80.9 ± 10.5	<0.0001
Hypertension (%)	498,952 (46.8)	221,449 (58.3)	86,834 (69.7)	29,484 (77.8)	<0.0001
Dyslipidemia (%)	380,152 (35.7)	173,055 (45.6)	58,176 (46.7)	21,034 (55.5)	<0.0001
DM duration ≥ 5 years (%)	306,461 (28.8)	65,192 (17.2)	55,941 (44.9)	12,921 (34.1)	<0.0001
Insulin use (%)	71,954 (6.8)	16,580 (4.4)	16,953 (13.6)	4135 (10.9)	<0.0001
OHA ≥ 3 (%)	143,862 (13.5)	39,644 (10.4)	23,172 (18.6)	6274 (16.6)	<0.0001
Statin use (%)	277,520 (26.1)	102,071 (26.9)	44,274 (35.6)	14,639 (38.6)	<0.0001
Laboratory results					
Fasting glucose (mg/dL)	144.1 ± 45.8	152.9 ± 47.1	138.9 ± 48.6	147.8 ± 52.3	<0.0001
Total cholesterol (mg/dL)	195.3 ± 43.6	211.9 ± 47.4	194.8 ± 45.1	209.1 ± 50.8	<0.0001
Triglyceride (mg/dL) ^†^	122.2 (122.1, 122.3)	232.7 (232.3, 233.0)	131.6 (131.3, 132.0)	231.7 (230.5, 233.0)	<0.0001
HDL-cholesterol (mg/dL)	53.1 ± 23.8	49.1 ± 32.1	53.1 ± 40.0	55.7 ± 62.4	<0.0001
Urine Protein					
Negative	983,624 (92.3)	335,195 (88.3)	103,959 (83.5)	30,280 (79.9)	<0.0001
Trace	32,872 (3.1)	15,754 (4.2)	5174 (4.2)	1808 (4.8)	
1+	30,976 (2.9)	17,327 (4.6)	7274 (5.8)	2732 (7.2)	
2+	13,212 (1.2)	8457 (2.2)	5212 (4.2)	1985 (5.2)	
3,4+	3728 (0.4)	2432 (0.6)	2354 (1.9)	893 (2.4)	

Abbreviations: NAFLD, nonalcoholic fatty liver disease; CKD, chronic kidney disease; BMI, body mass index; SBP, systolic blood pressure; DBP, diastolic blood pressure; DM, diabetes mellitus; OHA, oral hypoglycemic agents; HDL, high-density lipoprotein. Values are presented as mean ± standard deviation or median (range) for continuous variables and number (%) for categorical variables. ^†^ Geometric median (95% confidence interval).

**Table 2:** Risk of cardiovascular event according to CKD and NAFLD.

	Number of Subjects	Number of Events	Incidence Rate (1000 p-y)	Model 1	Model 2	Model 3	After IPTW
				Adjusted HR (95% CI)			
**Myocardial Infarction**							
No CKD/No NAFLD	1,065,190	24,759	3.35	1 (reference)	1 (reference)	1 (reference)	1 (reference)
No CKD/NAFLD	379,638	8617	3.28	1.18 (1.15–1.21)	1.16 (1.13–1.19)	1.21 (1.18–1.25)	1.27 (1.24–1.31)
CKD/No NAFLD	124,507	5752	6.85	1.45 (1.40–1.49)	1.33 (1.29–1.37)	1.33 (1.30–1.37)	1.18 (1.24–1.31)
CKD/NAFLD	37,897	1735	6.68	1.56 (1.49–1.64)	1.43 (1.36–1.50)	1.49 (1.42–1.57)	1.58 (1.50–1.67)
**Stroke**							
No CKD/No NAFLD	1,065,190	36,027	4.90	1 (reference)	1 (reference)	1 (reference)	1 (reference)
No CKD/NAFLD	379,638	11,468	4.39	1.18 (1.15–1.20)	1.14 (1.11–1.16)	1.21 (1.18–1.24)	1.33 (1.30–1.11)
CKD/No NAFLD	124,507	8340	10.04	1.28 (1.25–1.31)	1.19 (1.16–1.22)	1.19 (1.16–1.22)	1.08 (1.05–1.11)
CKD/NAFLD	37,897	2592	10.10	1.51 (1.45–1.57)	1.37 (1.32-1.43)	1.48 (1.41–1.54)	1.54 (1.47–1.62)
**Overall mortality**							
No CKD/No NAFLD	1,065,190	70,966	9.51	1 (reference)	1 (reference)	1 (reference)	1 (reference)
No CKD/NAFLD	379,638	19,468	7.35	1.09 (1.07–1.11)	1.10 (1.08–1.11)	1.54 (1.52–1.57)	1.69 (1.66–1.72)
CKD/No NAFLD	124,507	21,250	24.90	1.46 (1.44–1.48)	1.39 (1.37–1.42)	1.41 (1.39–1.43)	1.17 (1.15–1.19)
CKD/NAFLD	37,897	5293	20.04	1.41 (1.37–1.45)	1.37 (1.33–1.40)	2.00 (1.94–2.06)	2.13 (2.06–2.21)
**Cardiovascular mortality**							
No CKD/No NAFLD	1,065,190	12,490	1.67	1 (reference)	1 (reference)	1 (reference)	1 (reference)
No CKD/NAFLD	379,638	3349	1.26	1.14 (1.10–1.19)	1.09 (1.05–1.14)	1.47 (1.41–1.54)	1.57 (1.51–1.63)
CKD/No NAFLD	124,507	4659	5.46	1.67 (1.62–1.73)	1.55 (1.50–1.61)	1.57 (1.51–1.62)	1.34 (1.29–1.40)
CKD/NAFLD	37,897	1190	4.50	1.75 (1.65–1.85)	1.58 (1.49–1.68)	2.20 (2.07–2.35)	2.31 (2.15–2.49)

Abbreviations: p-y, person-year; CKD, chronic kidney disease; NAFLD, nonalcoholic fatty liver disease; HR, hazard ratio; CI, confidence interval; IPTW, inverse probability of treatment weighting. Model 1: adjusted for age and sex. Model 2: adjusted for age, sex, income, smoking, alcohol consumption, regular exercise, hypertension, dyslipidemia, glucose, diabetes duration, insulin use, and three or more oral hypoglycemic agents. Model 3: Model 2 plus adjusted for body mass index. The HRs were computed by weighted Cox proportional hazards models with IPTW.

**Table 3:** Risk of cardiovascular events in subjects with NAFLD.

	AAR	Number of Subjects	Number of Events	Incidence of Event (1000 Person-Years)	Model 1	Model 2
					Adjusted HR (95% CI)	
**Myocardial Infarction**						
Non-CKD	<0.8	180,271	3576	2.85	1 (reference)	1 (reference)
	<1.4	173,116	4266	3.56	0.96 (0.91,1.00)	1.01 (0.96, 1.05)
	≥1.4	26,251	775	4.42	1.08 (1.00, 1.17)	1.13 (1.04, 1.23)
CKD	<0.8	11,787	444	5.34	1.30 (1.18, 1.44)	1.21 (1.09, 1.34)
	<1.4	21,347	1047	7.18	1.41 (1.31, 1.51)	1.35 (1.26, 1.46)
	≥1.4	4763	244	7.93	1.43 (1.25,1.63)	1.38 (1.21, 1.58)
**Stroke**						
Non-CKD	<0.8	180,271	3889	3.11	1 (reference)	1 (reference)
	<1.4	173,116	6332	5.32	1.13 (1.08, 1.17)	1.14 (1.09, 1.19)
	≥1.4	26,251	1247	7.18	1.30 (1.22, 1.39)	1.29 (1.20, 1.37)
CKD	<0.8	11,787	564	6.82	1.25 (1.14, 1.37)	1.15 (1.05, 1.26)
	<1.4	21,347	1600	11.13	1.45 (1.36, 1.54)	1.38 (1.29, 1.46)
	≥1.4	4763	428	14.14	1.57 (1.42, 1.74)	1.50 (1.36, 1.67)
**Overall mortality**						
Non-CKD	<0.8	180,271	5452	4.32	1 (reference)	1 (reference)
	<1.4	173,116	10,646	8.81	1.24 (1.20, 1.29)	1.26 (1.22, 1.31)
	≥1.4	26,251	3370	19.02	2.22 (2.12, 2.32)	2.16 (2.06, 2.25)
CKD	<0.8	11,787	1015	12.03	1.40 (1.31,1.50)	1.32 (1.23, 1.41)
	<1.4	21,347	3185	21.46	1.75 (1.67, 1.84)	1.71 (1.63, 1.79)
	≥1.4	4763	1093	34.87	2.44 (2.28, 2.61)	2.36 (2.20, 2.52)
**Cardiovascular mortality**						
Non-CKD	<0.8	180,271	949	0.75	1 (reference)	1 (reference)
	<1.4	173,116	1864	1.54	1.19 (1.10, 1.29)	1.23 (1.14, 1.34)
	≥1.4	26,251	536	3.02	1.92 (1.72,2.14)	1.93 (1.73, 2.15)
CKD	<0.8	11,787	226	2.68	1.70 (1.47, 1.97)	1.56 (1.35, 1.81)
	<1.4	21,347	729	4.91	2.08 (1.88, 2.31)	2.00 (1.81, 2.22)
	≥1.4	4763	235	7.50	2.65 (2.28, 3.07)	2.57 (2.21, 2.98)

Abbreviations: AAR, AST to ALT ratio; CKD, chronic kidney disease; NAFLD, nonalcoholic fatty liver disease; HR, hazard ratio; CI, confidence interval. Model 1: Adjusted for age and sex. Model 2: Adjusted for age, sex, income, smoking, alcohol consumption, regular exercise, hypertension, dyslipidemia, glucose, diabetes duration, insulin use, three or more oral hypoglycemic agents, and body mass index.

**Table 4:** Risk of cardiovascular events in subjects with CKD according to proteinuria.

	Urine Protein	Number of Subjects	Number of Events	Incidence of Event (1000 p-y)	Multivariate Model HR (95% CI)	After IPTW HR (95% CI)
**Myocardial Infarction**						
Non-NAFLD	Negative	103,959	4286	6.04	1 (reference)	1 (reference)
	Trace	5174	258	7.45	1.17 (1.04–1.33)	1.14 (1.00–1.29)
	1+	7274	496	10.68	1.59 (1.44–1.75)	1.50 (1.36–1.65)
	2+	5212	438	13.64	2.01 (1.82–2.22)	1.77 (1.60–1.96)
	3,4+	2888	274	15.74	2.34 (2.06–2.65)	1.93 (1.70–2.19)
NAFLD	Negative	30,280	1219	5.82	1.19 (1.11–1.28)	1.23 (1.15–1.32)
	Trace	1808	81	6.51	1.29 (1.03–1.61)	1.30 (1.05–1.61)
	1+	2732	162	8.84	1.64 (1.40–1.93)	1.55 (1.32–1.82)
	2+	1985	169	13.20	2.43 (2.08–2.85)	2.29 (1.96–2.68)
	3,4+	1092	104	15.44	2.91 (2.38–3.55)	2.57 (2.11–3.13)
**Stroke**						
Non-NAFLD	Negative	103,959	6401	9.12	1 (reference)	1 (reference)
	Trace	5174	406	11.88	1.25 (1.13–1.38)	1.21 (1.09–1.34)
	1+	7274	644	14.01	1.40 (1.29–1.52)	1.30 (1.20–1.42)
	2+	5212	521	16.37	1.66 (1.52–1.82)	1.47 (1.34–1.61)
	3,4+	2888	368	21.65	2.33 (2.09–2.59)	1.92 (1.73–2.14)
NAFLD	Negative	30,280	18,979	9.16	1.25 (1.18–1.33)	1.30 (1.22–1.37)
	Trace	1808	138	11.25	1.49 (1.25–1.77)	1.57 (1.34–1.85)
	1+	2732	239	13.25	1.66 (1.46–1.90)	1.62 (1.42–1.85)
	2+	1985	182	14.36	1.81 (1.56–2.11)	1.68 (1.45–1.95)
	3,4+	1092	136	20.59	2.74 (2.30–3.25)	2.40 (2.04–2.84)
**Overall mortality**						
Non-NAFLD	Negative	103,959	16,002	22.24	1 (reference)	1 (reference)
	Trace	5174	1027	29.14	1.25 (1.29–1.34)	1.20 (1.23–1.28)
	1+	7274	1788	37.60	1.55 (1.48–1.63)	1.41 (1.34–1.48)
	2+	5212	1492	45.02	1.98 (1.87–2.09)	1.63 (1.54–1.72)
	3,4+	2888	941	52.43	2.59 (2.42–2.76)	1.92 (1.80–2.05)
NAFLD	Negative	30,280	3783	17.79	1.38 (1.32–1.43)	1.47 (1.41–1.53)
	Trace	1808	284	22.49	1.85 (1.68–2.03)	1.84 (1.64–2.05)
	1+	2732	482	25.74	1.33 (1.30–1.37)	1.89 (1.73–2.07)
	2+	1985	453	34.40	2.56 (2.32–2.81)	2.45 (2.24–2.69)
	3,4+	1092	291	41.76	3.53 (3.14–3.98)	3.09 (2.77–3.45)
**Cardiovascular mortality**						
Non-NAFLD	Negative	103,959	3469	4.82	1 (reference)	1 (reference)
	Trace	5174	235	6.67	1.34 (1.17–1.53)	1.28 (1.12–1.47)
	1+	7274	429	9.02	1.77 (1.60–1.96)	1.59 (1.43–1.76)
	2+	5212	321	9.69	2.07 (1.84–2.33)	1.69 (1.50–1.89)
	3,4+	2888	205	11.42	2.81 (2.44–3.25)	2.07 (1.80–2.38)
NAFLD	Negative	30,280	836	3.93	1.36 (1.24–1.48)	1.41 (1.30–1.53)
	Trace	1808	69	5.46	1.86 (1.46–2.37)	2.05 (1.65–2.54)
	1+	2732	120	6.41	2.09 (1.73–2.52)	2.04 (1.70–2.43)
	2+	1985	99	7.52	2.60 (2.12–3.19)	2.49 (2.06–3.00)
	3,4+	1092	66	9.47	3.77 (2.94–4.83)	3.30 (2.64–4.13)

Abbreviations: p-y, person-year; CKD, chronic kidney disease; NAFLD, nonalcoholic fatty liver disease; HR, hazard ratio; CI, confidence interval; IPTW, inverse probability of treatment weighting. Adjusted for age, sex, income, smoking, alcohol consumption, regular exercise, hypertension, dyslipidemia, glucose, diabetes duration, insulin use, three or more oral hypoglycemic agents, and body mass index.

## Data Availability

The dataset (NHIS-HEALS) supporting the conclusions of this article is available in the homepage of National Health Insurance Sharing Service (http://nhiss.nhis.or.kr/bd/ab/bdaba021eng.do, accessed on 1 August 2021). To gain access to the data, a completed application form, a research proposal, and the applicant’s approval document from the institutional review board should be submitted to and reviewed by the inquiry committee of research support of the NHIS. Currently, use of NHIS data is allowed only for Korean researchers.

## Supplementary Materials

The following supporting information can be downloaded at: https://www.mdpi.com/article/10.3390/biomedicines10061245/s1, Table S1: Risk of cardiovascular event according to CKD and NAFLD; Table S2: Risk of cardiovascular event according to CKD (defined by CKD-EPI equation) and NAFLD.
