# Supplementary material for: Combined Effects of Chronic Kidney Disease and Nonalcoholic Fatty Liver Disease on the Risk of Cardiovascular Disease in Patients with Diabetes
Source: Biomedicines. 2022 May 26;10(6):1245. doi: 10.3390/biomedicines10061245 (PMC9219946; doi:10.3390/biomedicines10061245)
Supplement: Supplementary file 1 [file biomedicines-10-01245-s001.zip › biomedicines-1669473-supplementary proof.pdf]

Supplementary Table S1. Risk of cardiovascular event according to CKD and NAFLD.

	Number of event	Incidence rate (1,000 p-y)	Model 1	Model 2	Model 3	Model 4
			Adjusted HR (95% CI)			
**Myocardial Infarction**						
No CKD/No NAFLD	24,759	3.35	1 (reference)	1 (reference)	1 (reference)	1 (reference)
No CKD/NAFLD	8,617	3.28	1.18 (1.15–1.21)	1.16 (1.13–1.19)	1.21 (1.18–1.25)	1.19 (1.16–1.23)
CKD/No NAFLD	5,752	6.85	1.45 (1.40–1.49)	1.33 (1.29–1.37)	1.33 (1.30–1.37)	1.33 (1.29–1.37)
CKD/NAFLD	1,735	6.68	1.56 (1.49–1.64)	1.43 (1.36–1.50)	1.49 (1.42–1.57)	1.47 (1.40–1.55)
**Stroke**						
No CKD/No NAFLD	36,027	4.90	1 (reference)	1 (reference)	1 (reference)	1 (reference)
No CKD/NAFLD	11,468	4.39	1.18 (1.15–1.20)	1.14 (1.11–1.16)	1.21 (1.18–1.24)	1.19 (1.16–1.22)
CKD/No NAFLD	8,340	10.04	1.28 (1.25–1.31)	1.19 (1.16–1.22)	1.19 (1.16–1.22)	1.18 (1.15–1.21)
CKD/NAFLD	2,592	10.10	1.51 (1.45–1.57)	1.37 (1.32-1.43)	1.48 (1.41–1.54)	1.45 (1.39-1.52)
**Overall mortality**						
No CKD/No NAFLD	70,966	9.51	1 (reference)	1 (reference)	1 (reference)	1 (reference)
No CKD/NAFLD	19,468	7.35	1.09 (1.07–1.11)	1.10 (1.08–1.11)	1.54 (1.52–1.57)	1.46 (1.44–1.48)
CKD/No NAFLD	21,250	24.90	1.46 (1.44–1.48)	1.39 (1.37–1.42)	1.41 (1.39–1.43)	1.35 (1.34–1.37)
CKD/NAFLD	5,293	20.04	1.41 (1.37–1.45)	1.37 (1.33–1.40)	2.00 (1.94–2.06)	1.79 (1.74–1.83)
**Cardiovascular mortality**						

No CKD/No NAFLD	12,490	1.67	1 (reference)	1 (reference)	1 (reference)	1 (reference)
No CKD/NAFLD	3,349	1.26	1.14 (1.10–1.19)	1.09 (1.05–1.14)	1.47 (1.41–1.54)	1.37 (1.47–1.43)
CKD/No NAFLD	4,659	5.46	1.67 (1.62–1.73)	1.55 (1.50–1.61)	1.57 (1.51–1.62)	1.52 (1.47–1.43)
CKD/NAFLD	1,190	4.50	1.75 (1.65–1.85)	1.58 (1.49–1.68)	2.20 (2.07–2.35)	2.00 (1.88–2.12)

Abbreviations: p-y, person-year; CKD, chronic kidney disease; NAFLD, nonalcoholic fatty liver disease; HR, hazard ratio; CI, confidence interval
Model 1: Adjusted for age and sex
Model 2: Adjusted for age, sex, income, smoking, alcohol consumption, regular exercise, hypertension, dyslipidemia, glucose, diabetes duration, insulin use and three or more oral hypoglycemic agents
Model 3: Model 2 plus adjusted for body mass index
Model 4: Model 3 plus adjusted for statin use

Supplementary Table S2. Risk of cardiovascular event according to CKD (defined by CKD-EPI equation) and NAFLD.

	Number of event	Incidence rate (1,000 p-y)	Model 1	Model 2	Model 3	Model 4
			Adjusted HR (95% CI)			
**Myocardial Infarction**						
No CKD/No NAFLD	24,759	3.35	1 (reference)	1 (reference)	1 (reference)	1 (reference)
No CKD/NAFLD	8,617	3.28	1.18 (1.15–1.21)	1.16 (1.13–1.19)	1.21 (1.18–1.25)	1.20 (1.16–1.23)
CKD/No NAFLD	5,752	6.85	1.41 (1.15–1.21)	1.31 (1.27–1.35)	1.31 (1.27–1.35)	1.31 (1.27–1.34)
CKD/NAFLD	1,735	6.68	1.52 (1.45–1.60)	1.40 (1.34–1.47)	1.46 (1.39–1.54)	1.45 (1.38–1.52)
**Stroke**						
No CKD/No NAFLD	36,027	4.90	1 (reference)	1 (reference)	1 (reference)	1 (reference)
No CKD/NAFLD	11,468	4.39	1.17 (1.15–1.20)	1.13 (1.11–1.16)	1.21 (1.18–1.24)	1.19 (1.17–1.32)
CKD/No NAFLD	8,340	10.04	1.24 (1.21–1.27)	1.15 (1.13–1.18)	1.16 (1.13–1.18)	1.15 (1.12–1.18)
CKD/NAFLD	2,592	10.10	1.48 (1.42–1.54)	1.35 (1.30-1.40)	1.45 (1.39–1.51)	1.43 (1.37-1.49)
**Overall mortality**						
No CKD/No NAFLD	70,966	9.51	1 (reference)	1 (reference)	1 (reference)	1 (reference)
No CKD/NAFLD	19,468	7.35	1.09 (1.07–1.10)	1.10 (1.08–1.12)	1.55 (1.52–1.58)	1.54 (1.51–1.57)
CKD/No NAFLD	21,250	24.90	1.43 (1.41–1.45)	1.37 (1.35–1.40)	1.39 (1.37–1.41)	1.38 (1.36–1.41)
CKD/NAFLD	5,293	20.04	1.39 (1.35–1.43)	1.35 (1.31–1.38)	1.97 (1.91–2.03)	1.95 (1.90–2.01)
**Cardiovascular mortality**						

No CKD/No NAFLD	12,490	1.67	1 (reference)	1 (reference)	1 (reference)	1 (reference)
No CKD/NAFLD	3,349	1.26	1.15 (1.11–1.20)	1.10 (1.06–1.15)	1.48 (1.42–1.55)	1.46 (1.40–1.53)
CKD/No NAFLD	4,659	5.46	1.65 (1.59–1.70)	1.54 (1.49–1.59)	1.55 (1.50–1.60)	1.54 (1.49–1.60)
CKD/NAFLD	1,190	4.50	1.70 (1.61–1.80)	1.54 (1.46–1.64)	2.15 (2.02–2.29)	2.12 (1.99–2.26)

Abbreviations: p-y, person-year; CKD, chronic kidney disease; NAFLD, nonalcoholic fatty liver disease; CKD-EPI, Chronic Kidney Disease Epidemiology Collaboration; HR, hazard ratio; CI, confidence interval

Model 1: Adjusted for age and sex

Model 2: Adjusted for age, sex, income, smoking, alcohol consumption, regular exercise, hypertension, dyslipidemia, glucose, diabetes duration, insulin use and three or more oral hypoglycemic agents

Model 3: Model 2 plus adjusted for body mass index

Model 4: Model 3 plus adjusted for statin use
